# Scientific Misinformation and Mistrust of COVID-19 Preventive Measures among the UK Population: A Pilot Study

**DOI:** 10.3390/vaccines11020301

**Published:** 2023-01-30

**Authors:** Alessandro Siani, Imogen Green

**Affiliations:** School of Biological Sciences, University of Portsmouth, Portsmouth PO1 2DY, UK

**Keywords:** COVID-19, scientific misinformation, preventive measures, fake news, survey, vaccines, social distancing, face masks

## Abstract

The popularisation of complex biomedical concepts brought about by COVID-19 has led to the rapid proliferation and diffusion of scientific misinformation, particularly among individuals with inadequate levels of scientific and digital literacy. A cross-sectional online survey of a UK population sample was conducted to address three key aims: to verify whether there is a correlation between participants’ belief in false information around COVID-19 and adherence to preventive measures; to investigate whether participants’ scientific misinformation and preventive behaviour are associated with their demographic characteristics; and to evaluate whether participants’ scientific misinformation and preventive behaviour can predict their likelihood of having contracted COVID-19. Non-parametric data analysis highlighted a strong negative correlation between participants’ belief in misinformation and their trust in preventive measures. Both variables were significantly associated with participants’ education levels, but not with their religious beliefs. Remarkably, neither science misinformation levels nor the trust in preventive measures were statistically associated with the likelihood of having contracted COVID-19. Taken together, these findings reinforce the urgency of ensuring that the population is equipped with adequate scientific literacy to enable them to evaluate the reliability of scientific information and recognise the importance of individual preventive behaviours to minimise community spread of infectious diseases.

## 1. Introduction

The COVID-19 pandemic has brought about dramatic upheaval to virtually all aspects of global human civilisation. In addition to the immediate health impact on patients and their families, the pandemic has caused immense economic loss, disrupted education and social interactions, limited travel and in some cases restricted personal liberties [1,2]. COVID-19 caused abrupt changes in how humans interact with each other, how they think and what they talk about; it brought scientific concepts (e.g., herd immunity threshold and R value) that used to be the prerogative of biomedical sciences experts to the forefront of news headlines and domestic conversations. The popularisation of such concepts put individuals unfamiliar with the biomedical field (and often with the rudiments of the scientific method itself) at an increased risk of being exposed to scientific misinformation and disinformation [3].

Misinformation is defined as “publicly available information that is misleading or deceptive relative to the best available scientific evidence or expertise at the time and that counters statements by actors or institutions who adhere to scientific principles without adding accurate evidence for consideration” [4]. In the present study, the word “misinformation” is used in its broader definition encompassing both *stricto sensu* misinformation (i.e., “information that is false but not created with the intention of causing harm”) and disinformation/malinformation (i.e., “deliberately created to harm a particular person, social group, institution, or country”) [5]. The choice to group deliberate and unintentional misinformation for the purposes of this paper reflects the study’s focus on the impact on the misinformed individuals, which is comparable regardless of the original source of misinformation [6].

Belief in incorrect or unsubstantiated health information has been shown to cause distrust in national health services and healthcare workers, as well as a decline in adherence to preventive behaviours [7]. Recently, a machine-learning analysis of 51,170 tweets unearthed a strong positive correlation between the volume of anti-mask tweets and the number of new COVID-19 cases within the US population [8]. Similarly, a randomised controlled trial involving 8001 participants revealed that exposure to online scientific misinformation is strongly associated with a decline in vaccination intent [9]. Even prior to the COVID-19 pandemic, vaccine hesitancy had been identified by the WHO as a major threat to global public health [10]. When the pandemic hit, a significant proportion of the eligible population declined or delayed their vaccinations disregarding the abundant clinical evidence of their safety and effectiveness [11,12]. In particular, parents and expecting mothers expressed considerable concerns regarding the safety of the vaccines for their children [13,14,15].

A growing body of literature investigates the relationship between socio-demographic characteristics and susceptibility to misinformation. Adherence to a religious belief has been associated with a decreased ability to discern misinformation in online sources, particularly in individuals holding dogmatic and fundamentalist views [16,17]. For example, religion has been identified as a key determinant of vaccine hesitancy both before and after the COVID-19 pandemic [17,18,19]. On the other hand, a recent study investigating factors associated with COVID-19 believability profiles (e.g., belief in conspiracy theories on the origin of the pandemic versus the evidence-backed zoonotic theory) highlighted that religious views “were marginally, and typically non-significantly, associated with COVID-19 belief profile membership” [20].

Higher levels of education are linked to improved literacy, numeracy, and analytical abilities. Thus, individuals with higher academic achievements may be more able to discriminate sources based on their reliability and are, therefore, less likely to accept scientific claims not backed by adequate empirical evidence [21,22]. However, there is conflicting evidence with regards to the correlation between education levels and belief in scientific misinformation. For example, vaccine hesitancy has been shown to be directly or inversely correlated with education levels depending on the surveyed population [23,24].

The objective of the present study was to elucidate the relationship between belief in commonly-held misconceptions about COVID-19 and trust to key preventive measures, and to investigate whether the two variables are differentially associated with religious belief, educational level, and likelihood of having contracted COVID-19. The study sought to verify three hypotheses. *H*_1_ was that belief in scientific misinformation on COVID-19 is associated with a decrease in adherence to preventive measures within a UK population sample. *H*_2_ was that participants’ educational levels and religious beliefs are associated with different levels of belief in scientific misinformation and attitude towards preventive measures. *H*_3_ is that participants’ belief in scientific misinformation and adherence to preventive measures are (positively and negatively, respectively) associated with their likelihood of having contracted COVID-19.

## 2. Methods

### 2.1. Ethical Approval

The present study was designed in compliance with the University of Portsmouth Ethics Policy and the UK Research Integrity Office Code of Practice for Research. The collection, analysis, and storage of data were performed in accordance with the General Data Protection Regulations (GDPR). Ethical approval (code BIOL-ETHICS #021-2021) was obtained by the investigators prior to the distribution of the survey. A disclaimer was used to inform participants of the purpose of the survey and its anonymous and voluntary nature. All participants were informed of their right to withdraw at any moment prior to the submission of their answers and that clicking the “submit” button indicated that they had read and agreed to the conditions of the study.

### 2.2. Survey Design and Distribution

A self-administered, cross-sectional online survey was designed using Google Forms. The survey was composed of 15 multiple-choice questions (see Supplementary Information, Table S1) designed to gather data on the participants’ demographic/educational background and quantify their belief in commonly held misconceptions on COVID-19 as well as their adherence to key preventive measures.

Adult participants residing in the UK were recruited using snowball convenience sampling by posting the survey link on Facebook and LinkedIn and encouraging participants to further share it on their own social media. The survey was carried out between December 2021 and February 2022. Participants who did not agree to the conditions laid out in the disclaimer as well as those under 18 years old or not resident in the UK were excluded from the study. As no contact information was collected from participants, no reminders or follow-up communication was sent to them or to those who refused to participate. All questions (aside from agreement to the disclaimer) were optional, so participants were allowed to skip any that they did not feel comfortable answering. Participation was entirely voluntary and no compensation (monetary or otherwise) was offered to participants.

A Scientific Misinformation Score and a Preventive Behaviour Score were calculated for each participant by assigning a numerical value (1 point for “Strongly Disagree” to 5 points for “Strongly Agree”) to their responses to a set of 5 and a set of 3 Likert-type questions (highlighted in Supplementary Information, Table S1). Therefore, the scores ranged from 5 to 25 points for the Scientific Misinformation Score and from 3 to 15 points for the Preventive Behaviour Score, in which a higher score represents higher levels of, respectively, belief in scientific misinformation and adherence to preventive measures. The 5 questions used to determine the Scientific Misinformation Score were developed based on online searches on commonly held misconceptions about the COVID-19 pandemic. The 3 questions used to determine the Preventive Behaviour Score were designed to quantify adherence to the preventive measures identified by the WHO as the top three most effective in minimising community spread, namely vaccination, social distancing, and mask wearing [25].

### 2.3. Statistical Analysis

Statistical analysis was carried out using IBM SPSS Statistics Version 28 (Armonk, NY, USA: IBM Corp). The reliability of the questions used to calculate the two scores was tested using Cronbach’s Alpha tests. The tests indicated a Cronbach’s Alpha value of 0.841 for the questions used to calculate the Preventive Behaviour Score and 0.721 for the questions used to calculate the Scientific Misinformation Score, indicating good internal consistency for both sets of questions.

Non-parametric statistical tests were used in the analysis due to the categorical nature of the data collected in the survey. Two-sided Kruskal–Wallis tests were used to compare median scores between groups of participants. In cases where a significant difference was observed, post-hoc pairwise comparisons were carried out using Dunn’s test, and the significance values adjusted using the Bonferroni correction to minimise the possibility of type I errors due to multiple comparisons. Kendall’s τ-b tests were performed to analyse correlations between the study variables. Multivariate linear regression analysis was performed for each dependent variable to address any potential confounding effect of the independent variables analysed in the study. A significance cut-off of *p* ≤ 0.05 was used for all tests.

The required sample size to meet the statistical constraints of the non-parametric tests was calculated using Cochran’s equation. A confidence level of 80%, which is considered by convention the standard to achieve a good statistical power in survey-based studies [26], is achieved with 164 participants with a margin of error of 5%.

## 3. Results

A total of 218 adult UK residents took part in the survey, corresponding to a confidence level of 85%. The participants’ demographic characteristics and their opinions on the UK government’s handling of the COVID-19 pandemic are available in the Supplementary Information (Table S2 and Figure S1, respectively).

The Kendall’s τ-b test revealed a significant negative correlation (τ_b_ = −0.409, *p* = 5.9 × 10^−15^) between the participants’ Scientific Misinformation Score and their Preventive Behaviour Score.

**Figure 1:**
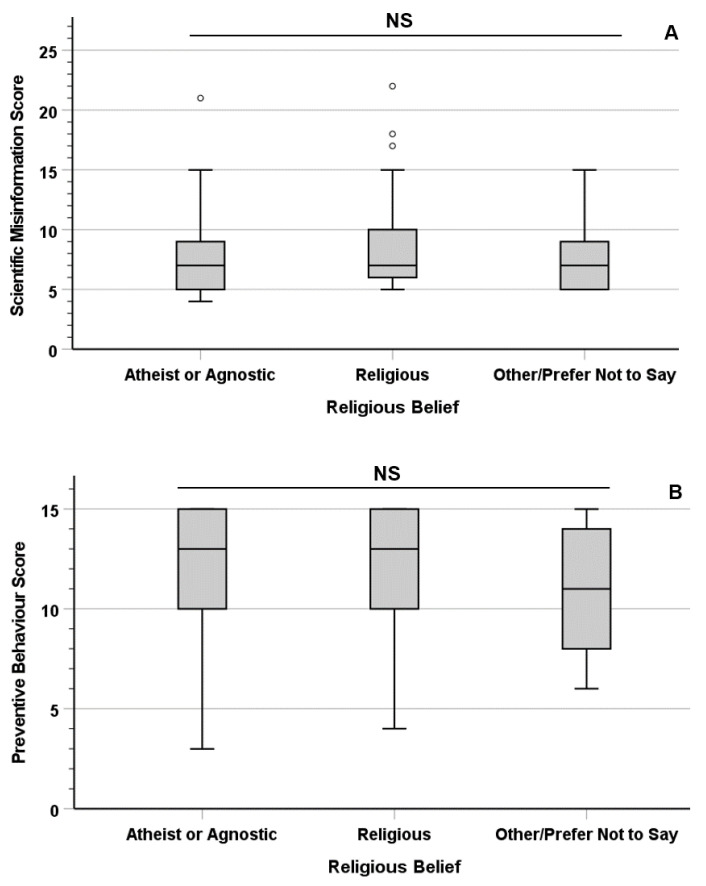
Association between the participants’ religious beliefs and their Scientific Misinformation Score (**A**) and Preventive Behaviour Score (**B**). Circles (°) indicate mild outliers.

The Kruskal–Wallis tests revealed no significant difference in the median Scientific Misinformation Score (χ^2^ = 2.223; df = 2; *p* = 0.329) and Preventive Behaviour Score (χ^2^ = 1.626; df = 2; *p* = 0.443), based on the participants’ religious beliefs (Figure 1).

**Figure 2:**
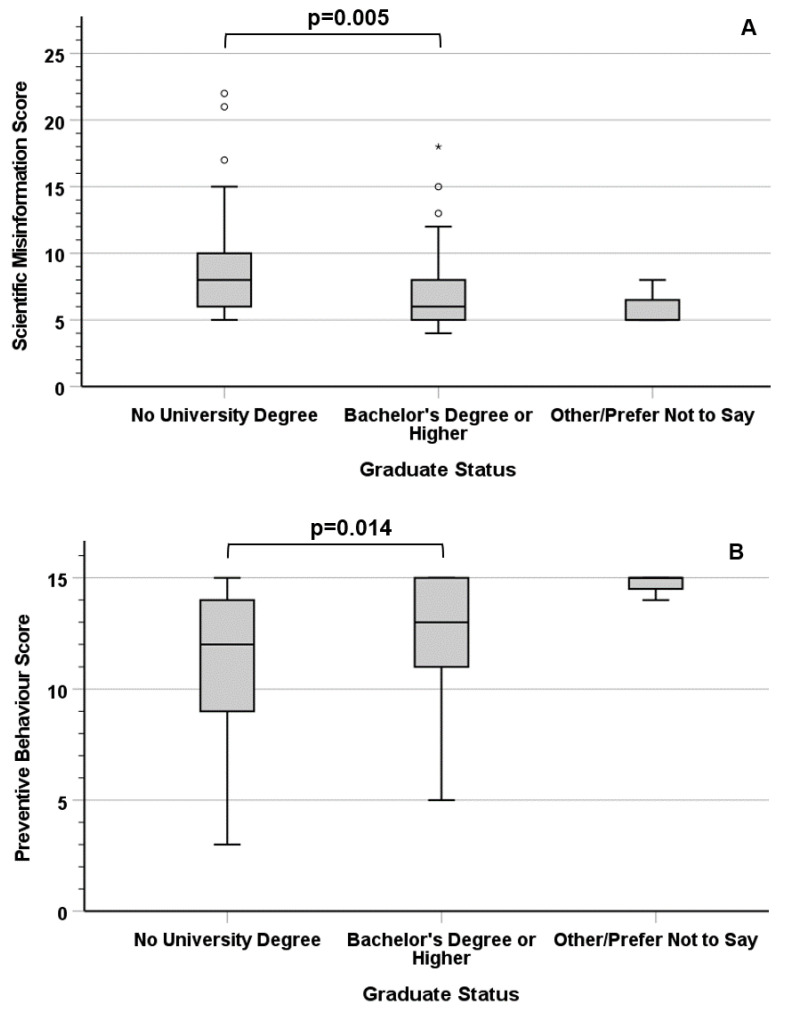
Association between the participants’ graduate status and their Scientific Misinformation Score (**A**) and Preventive Behaviour Score (**B**). Circles (°) indicate mild outliers, asterisks (*) indicate extreme outliers.

Statistically significant differences were observed in the median Scientific Misinformation Score (χ^2^ = 18.844; df = 2; *p* = 0.000081) and Preventive Behaviour Score (χ^2^ = 8.858; df = 2; *p* = 0.012) between respondents with different graduate status (Figure 2). Post-hoc pairwise tests revealed that participants with a university degree had a lower Scientific Misinformation Score (median: 6 vs. 8, *p* = 0.005) and a higher Preventive Behaviour Score (median: 13 vs. 12, *p* = 0.014) than those without a degree.

**Figure 3:**
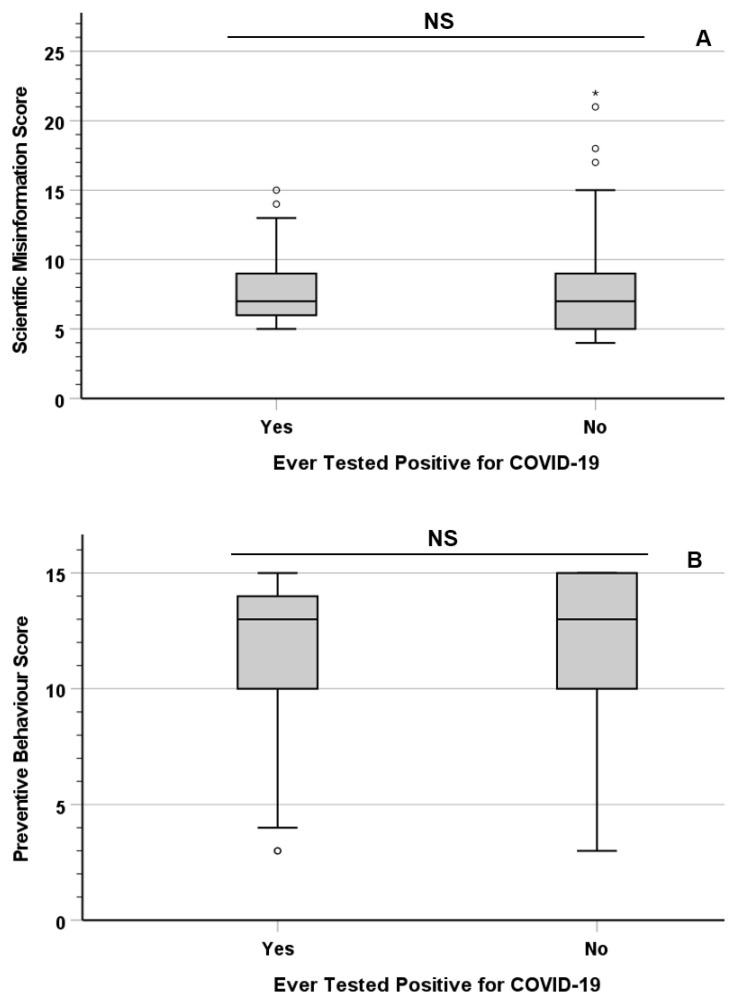
Scientific Misinformation Score (**A**) and Preventive Behaviour Score (**B**) for participants who tested positive for COVID-19 at least once and those who have not. Circles (°) indicate mild outliers, asterisks (*) indicate extreme outliers.

No significant differences were observed in the median Scientific Misinformation Score (χ^2^ = 0.756; df = 1; *p* = 0.384) and Preventive Behaviour Score (χ^2^ = 2.638; df = 1; *p* = 0.104) between the participants who tested positive for COVID-19 at least once and those who never had (Figure 3).

A multivariate regression analysis was performed to assess the potential confounding effects from any of the study variables. For all three dependent variables, multivariate regression confirmed the results of the non-parametric tests. The Scientific Misinformation Score was significantly correlated with the participants’ educational levels (*p* = 0.00005), but not with their religious beliefs (*p* = 0.105). Likewise, the Preventive Behaviour Score showed a significant correlation with educational levels (*p* = 0.0015) and non-significant correlation with religious beliefs (*p* = 0.327). The participants’ likelihood of testing positive for COVID-19 was not significantly associated with the participants’ educational levels (*p* = 0.929) nor religious beliefs (*p* = 0.408).

## 4. Discussion and Conclusions

The survey highlighted a strong negative correlation between the participants’ belief in COVID-19 misinformation and their trust in preventive measures, thereby confirming hypothesis *H_1_*. A recent latent class analysis of 20,000 UK adults reported that low compliance with preventive measures was associated with low confidence in the government, and that “individuals choose to comply with all guidelines, rather than some but not others” [27]. These observations are in line with the findings of the present study, in that mistrust in government-backed preventive measures is correlated with beliefs in “alternative” narratives regarding the pandemic. Similarly, a cross-sectional survey of a French population sample highlighted that individuals prone to conspiratorial beliefs are less willing to comply with government-driven preventive measures [28]. However, the same study reported that this behaviour is moderated by the motivation to protect oneself, i.e., that despite engaging in conspiratorial thinking, individuals may comply with preventive measures if they perceive themselves to be at risk of serious illness or death.

The second hypothesis of this study (*H*_2_) was that participants’ educational levels and religious beliefs are associated with different levels of belief in scientific misinformation and attitudes towards preventive measures. Hypothesis *H*_2_ was confirmed with regards to the existence of a positive association between the participants’ education levels and their trust in preventive measures, and a negative association between the participants’ education levels and their belief in scientific misinformation. While these findings are consistent with the outcomes of multiple previous studies [21,22,29], other studies report that educational levels are not significantly associated with belief in scientific misinformation and compliance with preventive measures [27,30]. The lack of a consensus regarding the impact of educational status on preventive behaviour and susceptibility to scientific misinformation indicates that their relation is confounded by other contextual variables; as different trends may therefore exist depending on the population being investigated, further studies are required to verify the existence and direction of this association.

The participants’ religious beliefs were not significantly associated with their belief in scientific misinformation nor with their trust in COVID-19 preventive measures; hypothesis *H_2_* was therefore rejected with regards to religious beliefs. While this finding is in apparent disagreement with previous studies reporting correlations between religion and scientific misinformation, it is important to remark that this effect appears to be mostly linked to dogmatic or fundamentalist views rather than religiosity *per se* [16,31]. Individuals holding extremist religious views have been shown to be a minority amongst British Christians and Muslims (representing the majority religious groups in both the present study sample and in the wider UK population), particularly in the younger generations [32,33].

The third and final hypothesis of the study (*H_3_*) was that participants’ belief in scientific misinformation and adherence to preventive measures are (positively and negatively respectively) associated with their likelihood of having contracted COVID-19. As no significant association was observed in that respect, the null hypothesis could not be rejected for *H*_3_. The observation that the likelihood of contracting COVID-19 is not statistically associated with trust in (and conceivably compliance with) preventive measures raises the alarming implication that, despite their scientifically proven efficacy in limiting viral transmission, individual preventive behaviours are not sufficient to stop infection within the community. While none of the preventive measures analysed in this study (mask-wearing, social distancing, and vaccination) are sufficient by themselves to protect an individual from contracting COVID-19, there is abundant evidence that their undeniable effectiveness in reducing community transmission hinges on their widespread adoption within a population [34,35,36,37]. Clear communication from governments and healthcare workers is pivotal in fostering adherence to preventive measures, even more so during a healthcare crisis such as a pandemic [38]. In particular, it is essential that categories at risk of hesitancy, such as parents and minorities with negative past experiences with healthcare providers, are provided with up-to-date information on the benefits of vaccinations and made aware of any potential risks (e.g., unwanted adverse effects) [39,40].

The generalisability of the outcomes of this study and their relevance to different populations are subject to some limitations. The non-random sampling strategy used in the study may result in the occurrence of selection bias, thereby providing less generalisable results compared to probability sampling. While the demographic features of the study population were largely comparable to the wider UK population in terms of religion, ethnicity, and educational levels, the sample was skewed towards a younger population range. This skewness, conceivably caused by the self-selection bias associated with online surveys distributed using social media, might impair the applicability of the findings to populations with different age profiles. Moreover, the data might be affected by biases typically associated with self-administered surveys, such as recall bias, social desirability bias, and non-response bias.

In summary, the survey discussed in this paper revealed a strong statistical correlation between scientific misinformation and mistrust of preventive measures in a UK population sample. The participants’ educational levels showed a direct association with their trust in preventive measures and an inverse association with their belief in scientific misinformation. On the other hand, no significant difference was observed based on the respondents’ religious beliefs (or lack thereof). Counterintuitively, the participants who reported having contracted COVID-19 at least once showed similar levels of scientific misinformation and trust in preventive measures as those who never tested positive, reinforcing the importance of the community-wide adoption of preventive behaviours and their promotion by governments and national health services.

## Data Availability

The data are available from the corresponding author upon reasonable request.

## Supplementary Materials

The following supporting information can be downloaded at: https://www.mdpi.com/article/10.3390/vaccines11020301/s1, Figure S1: Participants’ agreement with the statement “I believe that the UK government responded to the COVID-19 pandemic appropriately”; Table S1: questions used in the survey. Questions highlighted with an asterisk (*) were used for the calculation of the preventive behaviour score. Questions highlighted with two asterisks (**) were used for the calculation of the scientific misinformation score; Table S2: demographic characteristics of the study sample.
