# Supplementary material for: Scientific Misinformation and Mistrust of COVID-19 Preventive Measures among the UK Population: A Pilot Study
Source: Vaccines (Basel). 2023 Jan 30;11(2):301. doi: 10.3390/vaccines11020301 (PMC9966360; doi:10.3390/vaccines11020301)
Supplement: Supplementary file 1 [file vaccines-11-00301-s001.zip › vaccines-2100998-supplementary.pdf]

Table S1: questions used in the survey. Questions highlighted with an asterisk (*) were used for the calculation of the preventive behaviour score. Questions highlighted with two asterisks (**) were used for the calculation of the scientific misinformation score

1	What is your age?
2	What is the highest level of education you have completed?
3	What is your religious/spiritual belief?
4	What is your ethnicity?
5	Have you ever tested positive for COVID-19?
6	Which sources do you use to fact-check information you receive online about COVID-19, before forwarding to others?
7	I believe that the UK government responded to the COVID-19 pandemic appropriately.
8*	I believe face masks help reduce the spread of COVID-19.
9*	I believe social distancing measures help reduce the spread of COVID-19.
10*	I believe vaccinations help reduce the spread of COVID-19.
11**	The COVID-19 pandemic is caused by 5G mobile networks that spread the virus.
12**	Holding your breath for 10 seconds or more without coughing or discomfort means you are free from COVID-19.
13**	The COVID-19 vaccine impacts female fertility.
14**	Mask-wearing weakens the immune system.
15**	COVID-19 swab tests are invasive enough to cause damage to the brain.

Table S2: Demographic characteristics of the study sample

Age	Count	%	Ethnicity	Count	%
18-24	139	64.1	White	174	79.8
25-30	23	10.6	Black/African/Caribbean/Black British	5	2.3
31-40	11	5.1	Asian/Asian British	18	8.3
41-50	21	9.7	Mixed/Multiple Ethnic Groups	16	7.3
51-60	8	3.7	Other/Prefer Not to Say	5	2.3
60+	15	6.9			
**Religion**	**Count**	**%**	**Education**	**Count**	**%**
Atheist or Agnostic	105	48.2	No University Degree	125	57.3
Religious	96	44.0	Bachelor's Degree or Higher	90	41.3
Other/Prefer Not to Say	17	7.8	Other/Prefer Not to Say	3	1.4

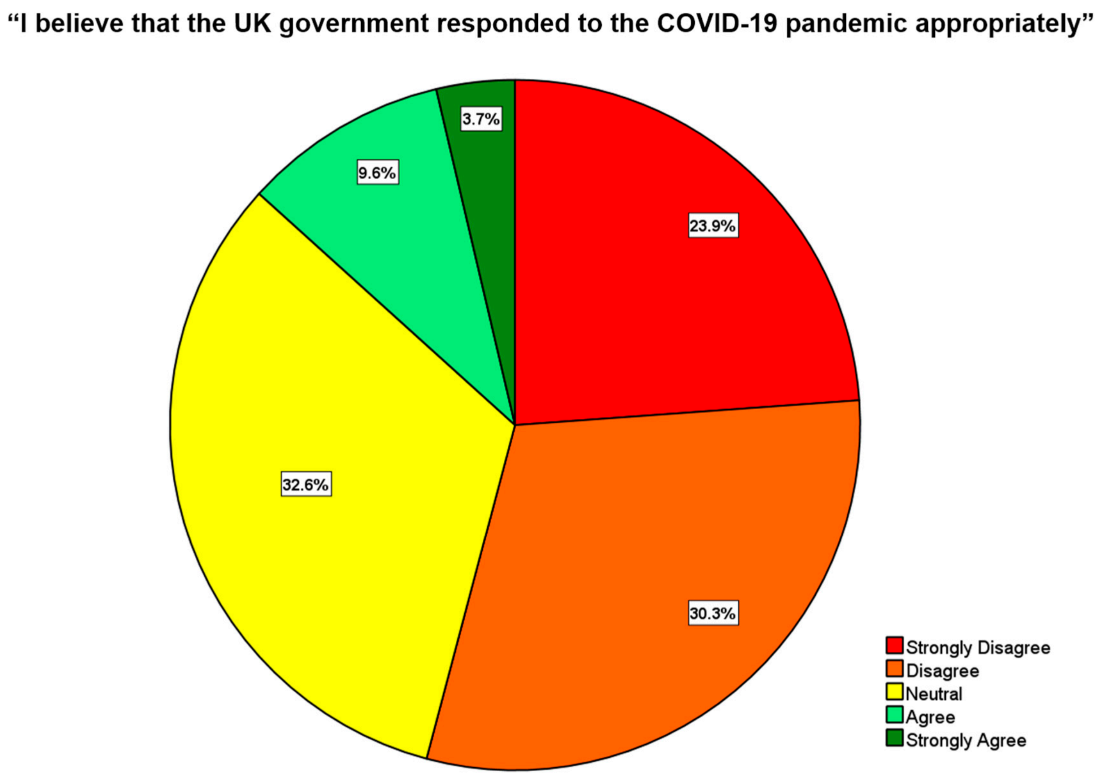

Figure S1: Participants’ agreement with the statement “I believe that the UK government responded to the COVID-19 pandemic appropriately”
